# Genotype and local environment dynamically influence growth, disturbance response and survivorship in the threatened coral, *Acropora cervicornis*

**DOI:** 10.1371/journal.pone.0174000

**Published:** 2017-03-20

**Authors:** Crawford Drury, Derek Manzello, Diego Lirman

**Affiliations:** 1 Marine Biology and Ecology Division, Rosenstiel School of Marine and Atmospheric Science, Miami, FL, United States of America; 2 Atlantic Oceanographic and Meteorological Laboratories, NOAA, Miami, FL, United States of America; Academia Sinica, TAIWAN

## Abstract

The relationship between the coral genotype and the environment is an important area of research in degraded coral reef ecosystems. We used a reciprocal outplanting experiment with 930 corals representing ten genotypes on each of eight reefs to investigate the influence of genotype and the environment on growth and survivorship in the threatened Caribbean staghorn coral, *Acropora cervicornis*. Coral genotype and site were strong drivers of coral growth and individual genotypes exhibited flexible, non-conserved reaction norms, complemented by ten-fold differences in growth between specific G-E combinations. Growth plasticity may diminish the influence of local adaptation, where foreign corals grew faster than native corals at their home sites. Novel combinations of environment and genotype also significantly affected disturbance response during and after the 2015 bleaching event, where these factors acted synergistically to drive variation in bleaching and recovery. Importantly, small differences in temperature stress elicit variable patterns of survivorship based on genotype and illustrate the importance of novel combinations of coral genetics and small differences between sites representing habitat refugia. In this context, acclimatization and flexibility is especially important given the long lifespan of corals coping with complex environmental change. The combined influence of site and genotype creates short-term differences in growth and survivorship, contributing to the standing genetic variation needed for adaptation to occur over longer timescales and the recovery of degraded reefs through natural mechanisms.

## Introduction

Coral reef ecosystems worldwide have experienced chronic degradation over the past century, with particularly drastic declines in recent decades. This deterioration is likely to continue as multiple natural and anthropogenic stressors interact on various spatial scales, impacting coral survivorship, growth, and reef structure and function [1]. Among the most impacted regions, the Western Atlantic has lost nearly 80% of coral cover since 1970 [2], driven partially by the loss of staghorn coral, *Acropora cervicornis*. This species plays a critical structural role as a reef-builder, creating habitat for fish and invertebrates and consolidating loose sediments and rubble [3]. *A*. *cervicornis* was formerly dominant throughout the Caribbean, but the impacts of White Band Disease and bleaching [4–6], storm damage [7] and the die-off of *Diadema antillarum* [8] have resulted in drastic population declines. While a few areas maintain healthy populations [9], other areas of formerly high abundance have lost > 95% of colonies [10, 11], causing an unprecedented shift in community structure [12]. In response to regional declines, *A*. *cervicornis* was listed as ‘threatened’ in 2006 under the United States Endangered Species Act [13] and is considered ‘critically endangered’ by the IUCN [14].

Understanding the intrinsic capacity for acclimatization and adaptive response to stress is vital for the long-term persistence of any species facing complex environmental change [15–17]. These processes are partially dependent on the influence of environment and genotype and serve to integrate their effects, which can be evaluated as phenotypic plasticity and local adaptation. Phenotypic plasticity is a characteristic of a trait (such as growth) in response to an environmental stimulus that provides the ability to produce different phenotypes from the same genotype [18, 19], facilitating a ‘better’ potential phenotype-environment match [20] across habitats. Recent evidence suggests that phenotypic plasticity may play a role coral’s response to change over intra- and transgenerational timescales, interacting with the effects of genetic adaptation across generations [21, 22]. These processes may be particularly important for long-lived individuals which must potentially cope with change over centuries [23].

Plasticity is evident in coral morphology [24–26] and is well known in plants, where it can be evolutionarily important [27, 28]. However, traits that influence the fitness of an organism, such as growth and survivorship, have remained mostly unexamined in the context of plasticity, though descriptions of these trends in single environments show genotypic differences [29, 30]. Phenotypic plasticity supports genetic diversity under stabilizing selection by enabling different individuals to maintain fitness by expressing advantageous phenotypes for a range of genotypes [31], which may be particularly important for sessile organisms that are unable to seek more hospitable conditions. Maintaining genetic diversity improves the potential to adapt to novel environmental changes like climate change [32] and increases resilience [33]. In *A*. *cervicornis*, the reliance on asexual reproduction means that branches (ramets) from a single individual can be long-lived [23] and may be transported into novel reef microenvironments [34], underscoring the importance of plasticity [26]. Further, during active restoration, plasticity allows corals to cope with changing or novel conditions, which may lead to expression of formerly cryptic genetic variation [35, 36]. Plasticity also dictates the range of environmental conditions where a given organism can reasonably be expected to survive and grow, critical for the persistence of threatened populations in changing climates [21, 37].

Another avenue for understanding the influence of environment and genotype is local adaptation, which is common in a variety of taxa [38] and has been examined in corals via transplantation [39, 40], laboratory experiments [41–43], and inferred from population structure [44, 45]. In marine organisms, local adaptation was historically considered unlikely because populations were thought to be demographically open, with high connectivity introducing new alleles [46]. However, population-genetic studies generally suggest more closed systems [47]. Restricted connectivity may be due to oceanographic patterns, larval behavior, life history, and post-settlement phenotype-environment mismatches [20, 37, 48], which allow for divergent selection in different habitats. On the Florida Reef Tract, recent evidence shows significant population structure among *A*. *cervicornis* populations and high diversity within individual reefs [49]. Thus, the fine-tuning of populations to the local environment via natural selection may be important for the survivorship of depleted coral populations.

In this study, we employ a common garden and reciprocal transplantation design to analyze local adaptation, phenotypic plasticity, reaction norms, and disturbance responses between genetically distinct *A*. *cervicornis* individuals. Local adaptation is examined here by the study of native vs. foreign populations of transplanted corals, emphasizing the comparison within individual reef habitats [50]. Our research focuses on small spatial scales with distances between sites as low as 2 km and a range of reef environments, treating habitats as points along a continuum rather than focusing on specific environmental clines. We also assess phenotypic responses across various environments using reaction norms, where comparisons can be made between individuals to reveal potentially ‘specialist’ or ‘generalist’ genotypes [51]. Using a variety of reefs (environments) and individuals (genotypes) also enables the description of the influence of each factor within the larger context the ecological response to active restoration in a threatened species. We test the hypotheses that: 1) Site and Genotype have significant effects on coral growth, survivorship and bleaching response; 2) there is a dynamic relationship between genotype and the environment where phenotypic plasticity produces variable reaction norms; and 3) local adaptation exhibited in native vs. foreign comparisons favors corals returned to their original collection site over corals sourced from other reefs. These data may contribute to increased efficiency and efficacy of active restoration while developing a better understanding of the roles of adaptation and acclimatization in coral reef health and resilience through environmental and genotypic effects.

## Materials and methods

### Experimental design

**Fig 1:**
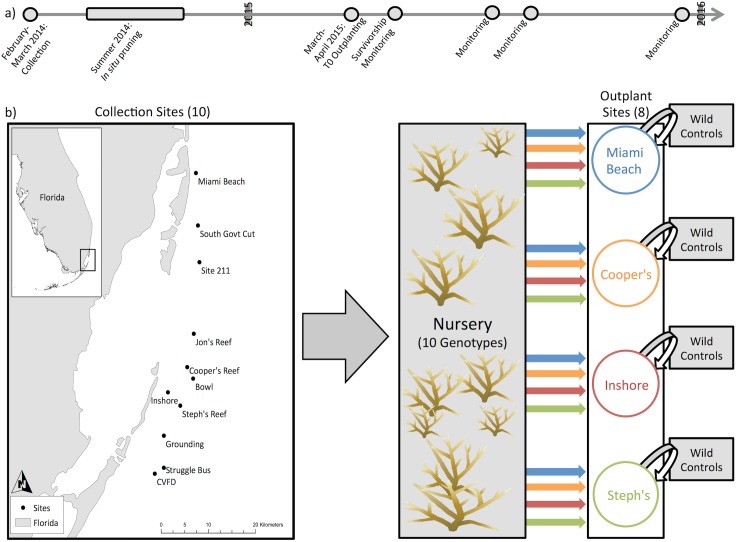
Experimental design and timeline. (a) Timeline for collection, propagation, outplanting and monitoring. (b) Fragments were collected from individual colonies at each collection site (n = 10 sites/genotypes) and transported to the nursery. After the nursery propagation phase, all genotypes were taken to 8 of 10 original collection sites (Government Cut, Site 211 excluded for logistical constraints), so that each site received its original collection, plus 9 other genotypes from each other reef. At each outplanting site, two colonies were collected as wild controls and installed in the plot with nursery transplants. Colors represent different genets coming from each reef, only 4 are presented here as an example.

We use a reciprocal transplantation experiment where donor reefs (original collection sites) receive native and foreign corals propagated in a common garden nursery. Under this fully crossed design, each site received every genotype from the common garden, including corals originally collected from that site and wild controls. The use of an *in situ* common garden serves as a genetic repository [52] and limits maternal effects by providing a single, consistent environment during propagation, helping to distinguish genetic differences from prior long-term acclimatization (origin site effects) [50]. During 2015, high summer temperatures caused bleaching among experimental corals, providing the additional opportunity to examine the role of GxE in stress response. As a result, growth (March-June), bleaching susceptibility (July-August), and post-bleaching recovery (December) were analyzed separately (Fig 1A).

### Nursery collections, outplanting & monitoring

Corals were collected from ten reefs in Miami-Dade County, Florida, USA during February-March 2014 (Fig 1B; Table A in S1 File). Site selection was designed to cover a large spatial range and the various habitat types of the Florida Reef Tract where *A*. *cervicornis* is present, including shallow and intermediate-depth sites (~2–11m depth), inshore-offshore sites, and patch reefs and consolidated hardbottom habitats (Table A in S1 File). At each site, 10–15cm branches were collected randomly from large, healthy donor colony and maintained in an *in situ* nursery until outplanting. Corals were haphazardly distributed among floating trees within the nursery and maintained until the beginning of the experiment (12–13 months). Collections were genotyped using Genotyping by Sequencing [53] and represent 10 unique individuals (Drury, unpublished data). In spring of 2015, ten nursery-grown corals from each genotype were fragmented from the nursery and outplanted to eight of the original collection sites (Fig 1B). All fragments outplanted from the nursery were approximately 10cm and only contained single branches when possible. There were no significant differences in initial size or number of branches between genotypes or sites. Two of the original collection sites were not used for outplanting due to logistical constraints (Table A in S1 File). Plots (approximately 2.5m x 3m) were created with nails on bare substrate approximately 25cm apart and were haphazardly populated with each genotype. Each plot was also populated with ten 10-cm fragments collected from each of two wild controls haphazardly selected at each site. Control corals from each genotype were outplanted on fixed-to-bottom platforms within the nursery. Measurements of total linear extension (LE), number of branches, condition, and survivorship were taken for every individual at installation and after 3, 4, 5 and 8 months. Corals were collected, maintained and outplanted under Biscayne National Park Permits BISC-2014-SCI-0018 and BISC-2015-SCI-0018 and Florida Fish and Wildlife Conservation Commission Special Activities License SAL-14-1086-SCRP.

### Genetic and physiological data

Immediately prior to outplanting, at least 7 replicate branches were collected for initial sampling of each genotype. Scrapings (1–2 polyps) were collected haphazardly from several locations on each fragment for all samples and were preserved in SDS (1% in DNAB) for *Symbiodinium* community analysis. Samples preserved in SDS were incubated at 65°C for two hours to prepare a RT-stable archive. This archive was sub-sampled and DNA extracted using a modified organic extraction protocol as in [54]. Symbiont clade was determined with qPCR using actin primers and probes to investigate the impact of initial *Symbiodinium* community on coral growth and survivorship as in [55]. The remaining skeleton and tissue were frozen at -80°C for analyzing lipid content. Coral tissue was removed from the skeleton of each sample using an airgun with freshly filtered seawater (0.35μm), homogenized, vacuum-filtered onto a glass fiber filter (Wattman GF/A), and frozen at -80°C for lipid extraction. Total lipid extraction followed the protocol of [56]. Skeletal sample surface area and volume were calculated from 3D scans as in [57]. Fragments were scanned using a white light 3D scanner (HDI Advance R2, 3D3 Solutions) using two 2-megapixel monochrome cameras and calibrated with a 5mm calibration board. Each sample was scanned every 20° rotation and scans were aligned and compiled into a single model using Flexscan 3D software. Models were trimmed, resampled to reduce holes in the model, and surface area and volume were measured using Leios 2 software. Skeletons were then dried overnight at 60°C and weighed to calculate density.

### Environmental data

Water temperature was recorded using logger pendants installed on a single nail within the plot (Onset Corp #UA-002-08; Massachusetts, USA) at hourly intervals. At each monitoring interval, seawater collections were made at depth in the center of the plot with a 500mL borosilicate bottle and poisoned with 200μL mercuric chloride for determination of carbonate chemistry. Temperature was measured at the time of water collections using a YSI Inc. YSI-85. Salinity was measured from water samples with a densitometer. Water samples were analyzed as in [57]. Briefly, dissolved inorganic carbon (Apollo SciTech AS-C3) and total alkalinity (Apollo SciTech AS-ALK2) were determined and input into CO2SYS [58] to calculate aragonite saturation state (Ω_arag_) using the dissociation constants of [59] as refit by [60] and [61] for boric acid. In September 2015, comparative light readings were taken for several days at each of four sites using PAR loggers (WETLab ECO-PAR; Oregon, USA). The loggers were installed immediately adjacent to plots and recorded total PAR (μmol/m^2^/sec) at 15-min intervals. Due to logistical constraints, light data were collected during a one-week deployment at paired sites (‘Cooper’s’ and ‘Inshore’) and over a subsequent 6-day deployment at a separate pair of sites (‘Struggle Bus’ and ‘CVFD’). Due to fouling, only the first 4 days of each deployment were used for analyses. Daytime light levels (9 am-5 pm) used in this study were averaged across all 4 days.

### Statistical analyses

All analyses were performed in JMP Pro 11.0.0. Coral growth over the first 3 months was converted to a daily growth rate and normalized to initial number of branches [29]. Data were transformed when necessary to meet homoscedasticity and normality assumptions of parametric tests and non-parametric tests were used when assumptions were not met. Only growth data over the first three months of the experiment were used, as subsequent bleaching stress had a substantial impact on growth. A 2-way ANOVA was used to examine main factors and subsequent 1-way ANOVAs were used within sites and within genotypes. One-way ANOVA was used to test differences within genotypes (across sites) and within sites (across genotypes). To evaluate growth in the context of phenotypic plasticity, average growth rate for each genotype was transformed into rank relative to the average growth at each site (above, at, below) and each G-E combination was classified. These values represent the reaction norm, or the spectrum of growth rates (phenotypes) for a given genotype across environments, which can vary in shape, magnitude, or both. To examine bleaching susceptibility and recovery, conditional distributions were tested using χ^2^ tests. Mortality was examined using a Cox-Proportional-Hazard model with Site, Genotype and interaction as factors. To test local adaptation, t-tests were used within each site with growth pooled between native corals from that site (native and control genotypes) and corals from foreign sites (all other genotypes). T-tests were used to compare growth, bleaching and mortality between ‘high’ and ‘low’ light regimes and ‘warm’ and ‘cool’ temperature regimes. Bonferroni corrected p-values were used for within site, within genotype and site-specific local adaptation comparisons.

## Results

### Overall growth

**Fig 2:**
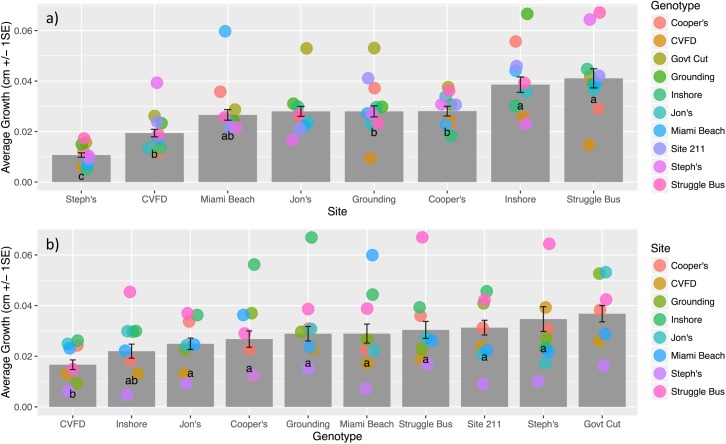
Growth of pooled corals by treatment. (a) Average LE (cm/day +/- 1 S.E.) at each site with all genotypes pooled. Due to logistical constraints, the fully crossed design did not include the site Jon’s Reef, which is included for representation but was not included in 2-way ANOVA analysis. Letters represent significant difference levels in log transformed data (Tukey's HSD, p<0.05), but untransformed data is presented here. Jittered points represent the growth of each genotype within that site and are color coded by genotype. (b) Average LE (cm/day +/- 1 S.E.) of each genotype, pooled across all sites. Due to logistical constraints, the fully crossed design did not include the genotype Government Cut, which is included for representation but was not included in 2-way ANOVA analysis. Letters represent significant difference levels in log transformed data (Tukey's HSD, p<0.05), but untransformed data is presented. Jittered points represent the growth at each site for that genotype and are color coded by site.

930 coral fragments representing ten nursery genotypes plus site controls were outplanted to eight reefs. One site (Jon’s Reef) did not receive one genotype (Cooper’s, due to limited fragment availability) or wild controls (no wild colonies were found on site). To test a fully crossed design, one site (Jon’s Reef) and one genotype (Government Cut) were eliminated from the two-way ANOVA, chosen to preserve a balance in number of treatments by site (n = 7) and genotype (n = 9). Growth rate (Linear Extension, LE) ranged from 0.005–0.067 cm/day across all genotypes and sites, with an experiment-wide average of 0.026 ± 0.001 cm/day (mean± 1 S.E.). A two-factor ANOVA showed a significant effect of Genotype (F_(8,356)_ = 4.743, p<0.001) and Site (F_(6,356)_ = 18.316, p<0.001), but no significant interaction effects (F_(48,356)_ = 1.222, p = 0.159). The ANOVA explained 41% of overall variance, with Site and Genotype explaining ~19% and ~6% respectively. Post-hoc tests identified three significantly different LE classes among the 7 reef sites (Fig 2A; Tukey’s HSD, p< 0.05) and two classes among the 9 coral genotypes (Fig 2B; Tukey’s HSD, p< 0.05).

### Growth by site

There were significant differences between Sites, with over a three-fold difference in LE, ranging from 0.010 ± 0.001 cm/day at Steph’s Reef to 0.044 ± 0.004 cm/day at Struggle Bus (Fig 2A). There were significant differences in LE between genotypes within a site at 3 of eight of reefs (Table 1; S2 File).

**Table 1:** Growth of specific genotype x environment combinations at each site.

		Site								
		CP	CVFD	GR	IN	JN	MB	ST	SB	NUR
**Genet**	**Control 1**	0.017	0.009	0.024	0.012	**NA**	0.02	0.006	0.018	**NA**
	**Control 2**	0.049	0.015	0.025	0.01	**NA**	0.016	0.007	0.067	**NA**
	**Cooper's**	0.023	0.012	0.037	0.056	**NA**	0.036	0.012	0.029	0.031
	**CVFD**	0.024	0.013	0.009	0.026	0.025	0.023	0.006	0.015	0.014
	**Govt Cut**	0.038	0.026	0.053	**NA**	0.053	0.029	0.016	0.042	0.022
	**Grounding**	0.031	0.023	0.030	0.067	0.031	0.024	0.015	0.039	0.012
	**Inshore**	0.018	0.013	0.030	0.030	0.030	0.022	0.005	0.045	0.025
	**Jon's**	0.034	0.013	0.023	0.036	0.024	0.025	0.009	0.037	0.011
	**Miami Beach**	0.023	0.017	0.027	0.044	0.022	0.060	0.007	0.039	0.014
	**Site 211**	0.031	0.024	0.041	0.046	0.021	0.022	0.009	0.042	0.017
	**Steph's**	0.031	0.039	0.027	0.023	0.017	0.022	0.010	0.064	0.019
	**Struggle Bus**	0.036	0.019	0.023	0.039	0.027	0.026	0.017	0.067	0.018
	**p-value**	0.03	**§0.01**	0.14	*0.01	*0.01	0.74	0.01	0.03	0.01

Average LE (cm/day) of each genotype (row) within each site (column). Significant differences in genotypes within sites are denoted with *(One-Way ANOVA) or §(Kruskal-Wallis) at the bottom of each column using p-values with a Bonferroni correction. Significant differences occurred at 3 of 8 sites. Colors reflect values above (green), at (yellow) or below (red) median LE within each site. Control corals were collected at the time of outplanting from each site independently, thus they do not represent the same genotype across columns. Site abbreviations: CP = Cooper’s, CVFD = CVFD, GR = Grounding, IN = Inshore, JN = Jon’s, MB = Miami Beach, ST = Steph’s, SB = Struggle Bus, NURS = Nursery.

### Growth by genotype

There were significant differences in LE among genotypes (Fig 2B), with growth rates ranging from 0.016 ± 0.002 (mean cm/day ± 1 S.E.) for CVFD to 0.036± 0.003 cm/day for Government Cut. When each genotype’s LE was examined among sites, there were significant differences within 7 of 10 genotypes (Table 2; S2 File).

**Table 2:** Growth of specific genotype x environment combinations for each genotype.

		Site									
		CP	CVFD	GR	IN	JN	MB	ST	SB	NUR	p-value
**Genet**	**Control 1**	0.017	0.009	0.024	0.012	**NA**	0.02	0.006	0.018	**NA**	**NA**
	**Control 2**	0.049	0.015	0.025	0.01	**NA**	0.016	0.007	0.067	**NA**	**NA**
	**Cooper's**	0.023	0.012	0.037	0.056	**NA**	0.036	0.012	0.029	0.031	*0.01
	**CVFD**	0.024	0.013	0.009	0.026	0.025	0.023	0.006	0.015	0.014	0.03
	**Govt Cut**	0.038	0.026	0.053	**NA**	0.053	0.029	0.016	0.042	0.022	*0.01
	**Grounding**	0.031	0.023	0.03	0.067	0.031	0.024	0.015	0.039	0.012	0.01
	**Inshore**	0.018	0.013	0.03	0.03	0.03	0.022	0.005	0.045	0.025	*0.01
	**Jon's**	0.034	0.013	0.023	0.036	0.024	0.025	0.009	0.037	0.011	**§0.01**
	**MB**	0.023	0.017	0.027	0.044	0.022	0.06	0.007	0.039	0.014	0.16
	**Site 211**	0.031	0.024	0.041	0.046	0.021	0.022	0.009	0.042	0.017	**§0.03**
	**Steph's**	0.031	0.039	0.027	0.023	0.017	0.022	0.01	0.064	0.019	*0.01
	**SB**	0.036	0.019	0.023	0.039	0.027	0.026	0.017	0.067	0.018	*0.01

Average LE (cm/day) of each genotype (row) at every site (column). Significant differences between sites (excluding nursery) for a given genotype are denoted with *(One-Way ANOVA) or § (Kruskal-Wallis) to the right of the row using p-values with a Bonferroni correction. Significant differences between sites occur in 7 of 10 genotypes. Colors reflect values above (green), at (yellow) or below (red) median LE within each genotype. Lack of significance of Miami Beach corals is likely due to lower sample size at some sites (high mortality). Control corals were collected at the time of outplanting from each site independently, thus they do not represent the same genotype across columns and no averages are presented. Site abbreviations: CP = Cooper’s, CVFD = CVFD, GR = Grounding, IN = Inshore, JN = Jon’s, MB = Miami Beach, ST = Steph’s, SB = Struggle Bus, NURS = Nursery.

### Local adaptation

**Fig 3:**
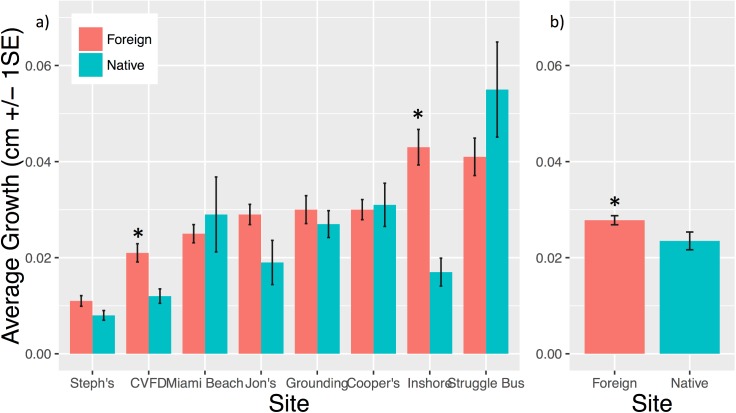
Local adaptation comparisons of growth. (a) Average growth (cm/day +/- 1 S.E.) of local and foreign corals at each site. Significant differences between local and foreign genotypes within a given site are denoted by *(t-test p<0.05). Higher growth in foreign corals suggests lack of local adaptation among native genotypes at any given reef. (b) Average growth of native and foreign corals pooled across all sites, significant differences between native and foreign are denoted by *(t-test p<0.05). Local corals were wild controls plus corals originally collected from each site before propagation in the nursery. Foreign corals are genotypes collected from all other sites.

LE was significantly higher in foreign than in native corals at two sites (Fig 3A): CVFD (t-test p = 0.001) and Inshore (t-test p<0.001). Five transplant sites showed a trend of higher LE of foreign corals and three sites exhibited a trend of higher LE of native corals, though none of these differences were significant (Fig 3A; Table B in S1 File; S2 File). Pooled (across sites) foreign genotype LE was significantly higher than LE of pooled native genotypes (Fig 3B; t-test p = 0.021). During the initial period, mortality of native genotypes was higher than that of foreign genotypes at 3 sites, however, after three months there was no significant difference in percent survivorship among pooled native and foreign corals (89% and 85%, respectively, t-test p = 0.675). At the conclusion of the experiment, there was no significant difference in survivorship of native and foreign corals (14% and 11%, respectively, t-test p = 0.629).

### Phenotypic plasticity and reaction norms

There were three genotypes with LE at or above the median for >75% of sites, representing fast-growing corals (Government Cut, Grounding, and Struggle Bus; Table 1). Conversely, two genotypes (CVFD and Jon’s) had LE at or below the median for at least 75% of sites, representing slow-growing corals. Regardless of growth rate, genotypes were further divided into ‘generalists’, which have relatively flat reaction norms and lower variability in growth rate between sites, and ‘specialists’, which have widely fluctuating LE dependent on the environmental conditions at a given site. For example, the Government Cut genotype has above median LE at every site, while the Struggle Bus genotype is at or above the median at all except one location (Table 1). These genotypes may be successful over a wide range of sites. Conversely, some genotypes are ‘specialists’: Jon’s, Miami Beach and Inshore fluctuate above or below the median at different sites. A single genotype (Steph’s) represents especially high specificity; it is the only individual to show the highest ranked LE rate within one site (CVFD) and the lowest LE within another site (Jon’s). Importantly, relative growth rates are never conserved between genotypes across all sites and many genotypes exhibit multiple pairwise reciprocal changes in ranking between sites (Table 2). For example, Jon’s and Inshore genotypes only share a relative ranking (above or below median) at a single site (CVFD). Nursery growth (Table 1) is not a good predictor of growth at any site, as relative growth rank within the nursery does not match any of the outplant sites. The dependence on both site and genotype is further highlighted by differential growth rates in 70% of genotypes between sites (Table 2), with at least a two-fold difference in growth rate between the fastest and slowest sites for every genotype. Faster growing coral genotypes do not have a higher range of LE (F_(1,6)_ = 1.84, p = 0.2118, R^2^ = 0.187), however, sites with a wider range of coral growth showed a higher average LE (F_(1,6)_ = 10.175, p = 0.019 R^2^ = 0.629).

### Physiological data

All coral genotypes contained *Symbiodinium* from Clade A at the time of transplantation, with no detectable background levels of clades B, C, or D. Total lipids ranged from 1.79–2.42 mg/cm^2^ and were not significantly different between genotypes (ANOVA, F_(9,42)_ = 0.7962, p = 0.621). There was a weak positive relationship between lipid content and LE (r^2^ = 0.246, p = 0.144). There was a significant difference in skeletal density between genotypes, ranging from 0.631–0.848 g/cm^3^ (Kruskal-Wallis, H = 20.802, p = 0.014) and a strong negative relationship between LE and skeletal density (r^2^ = 0.838, p = 0.001).

### Environmental data

There were no significant differences in aragonite saturation state (Ω_arag_) between sites (Table C in S1 File; S2 File), or a relationship between Ω_arag_ and LE (regression p>0.05). Light regimes were significantly different among sites (Fig A in S1 File; S2 File; ANOVA, F_(3,131)_ = 23.273, p<0.001). LE for all genotypes combined was significantly higher at ‘low’ light sites (Struggle Bus and Inshore) than at ‘high’ light sites (Cooper’s and CVFD; t-test p<0.001). Average daily temperature for the growth period (March to June) ranged from 27.2–28.8°C at different sites (Table A in S1 File; Fig B in S1 File). There was a significant difference in daily average temperature based on site (ANOVA, F_(7,739)_, p<0.001), but no temperature metrics predicted growth (regression, p>0.05).

### Bleaching impacts

Bleaching was not observed during the growth period (March-June). During July and August, average temperatures ranged from 30.6–31.1°C and were significantly different between sites (ANOVA, F_(7,423)_ = 10.55, p<0.001), with two discrete site groupings identified based on temperature, though average differences between sites was <0.5°C. The ‘hot’ sites (CVFD, Inshore, Steph’s, and Grounding) average 2.8 m deep while ‘cool’ sites (Jon’s, Miami Beach, Struggle Bus and Cooper’s) average 5.8 m. During July, sites experienced significantly different levels of bleaching prevalence, which ranged from 13%-47% of colonies (Table 3; χ^2^ = 37.1, df = 7, p<0.01). At this time, prevalence was also significantly different based on genotype (χ^2^ = 68.7, df = 9, p<0.01) with 9%-53% of corals per genotype bleaching (Table 3). During July, there were no significant differences in bleaching prevalence at ‘hot’ and ‘cool’ sites (t-test, p = 0.529). There was higher bleaching at ‘low’ light sites than ‘high’ light sites during July (34% vs. 17%, respectively).

**Table 3:** Bleaching prevalence of outplanted corals in July.

		Site								
		CP	CVFD	GR	IN	JN	MB	ST	SB	Average
**Genet**	**Control1**	0%	20%	60%	22%	**NA**	0%	11%	0%	
	**Control2**	25%	40%	30%	33%	**NA**	17%	43%	0%	
	**Cooper's**	60%	11%	56%	50%	**NA**	50%	29%	50%	44%
	**CVFD**	22%	10%	57%	60%	20%	88%	20%	50%	41%
	**Govt Cut**	30%	0%	70%	**NA**	40%	11%	22%	50%	32%
	**Grounding**	25%	0%	10%	0%	11%	13%	0%	14%	9%
	**Inshore**	0%	0%	20%	25%	20%	0%	0%	33%	12%
	**Jon's**	40%	30%	89%	80%	50%	38%	44%	50%	53%
	**Miami Beach**	30%	0%	50%	33%	25%	25%	25%	0%	24%
	**Site 211**	0%	40%	89%	100%	60%	33%	43%	20%	48%
	**Steph's**	10%	10%	29%	0%	50%	0%	29%	14%	18%
	**Struggle Bus**	22%	0%	0%	33%	0%	20%	0%	17%	12%
	**Average**	22%	13%	47%	40%	31%	24%	22%	25%	

Percent bleaching prevalence of live corals of each genotype (row) at each site (column) for July. Blank cells represent combinations of site and genotype with 100% mortality (i.e. no live corals for bleaching percentage calculations). Color scales represent bleaching prevalence, with darker cells representing higher bleaching. Average column (far right) represents the average bleaching for each genotype (row). Average row (bottom) represents average bleaching for each site (column). In July, both site and genotype were significantly different from even distribution of bleaching. In August, only site was significant, as temperatures were so high they had likely overwhelmed any genetic differences. Control corals were collected at the time of outplanting from each site independently, thus they do not represent the same genotype across columns and no averages are presented. Site abbreviations: CP = Cooper’s, CVFD = CVFD, GR = Grounding, IN = Inshore, JN = Jon’s, MB = Miami Beach, ST = Steph’s, SB = Struggle Bus, NURS = Nursery.

During August, bleaching prevalence was significantly different among sites (Table 4; χ^2^ = 25.6, df = 7, p<0.05), with 63% -99% of colonies bleached within sites. Unlike in July, there were no differences in bleaching prevalence among genotypes as temperature stress intensified in August (Table 4). During this timepoint, bleaching prevalence at ‘hot’ and ‘cool’ was not different (t-test, p = 0.281), but bleaching was higher at ‘high’ versus ‘low light’ sites (91% vs. 67%, respectively).

**Table 4:** Bleaching prevalence of outplanted corals in August.

		Site								
		CP	CVFD	GR	IN	JN	MB	ST	SB	Average
**Genet**	**Control1**	89%	67%	71%	38%	**NA**	57%	100%	100%	
	**Control2**	100%	100%	86%	63%	**NA**	100%	100%	100%	
	**Cooper's**	100%	88%	89%	**—**	**NA**	100%	86%	50%	85%
	**CVFD**	100%	100%	50%	100%	100%	80%	100%	67%	87%
	**Govt Cut**	75%	100%	100%	**NA**	100%	50%	100%	75%	86%
	**Grounding**	100%	90%	100%	100%	86%	**—**	100%	25%	86%
	**Inshore**	100%	75%	100%	80%	44%	100%	100%	75%	84%
	**Jon's**	70%	90%	100%	80%	86%	63%	100%	100%	86%
	**Miami Beach**	90%	100%	33%	100%	100%	50%	100%	25%	75%
	**Site 211**	100%	88%	50%	100%	100%	**—**	100%	80%	88%
	**Steph's**	100%	100%	67%	67%	100%	67%	100%	40%	80%
	**Struggle Bus**	78%	90%	89%	100%	100%	67%	100%	20%	80%
	**Average**	92%	91%	78%	83%	91%	73%	99%	63%	

Percent bleaching prevalence of live corals of each genotype (row) at each site (column) for August. Blank cells represent combinations of site and genotype with 100% mortality (i.e. no live corals for bleaching percentage calculations). Color scales represent bleaching prevalence, with darker cells representing higher bleaching. Average column (far right) represents the average bleaching for each genotype (row). Average row (bottom) represents average bleaching for each site (column). In July, both site and genotype were significantly different from even distribution of bleaching. In August, only site was significant, as temperatures were so high they had likely overwhelmed any genetic differences. Control corals were collected at the time of outplanting from each site independently, thus they do not represent the same genotype across columns and no averages are presented. Site abbreviations: CP = Cooper’s, CVFD = CVFD, GR = Grounding, IN = Inshore, JN = Jon’s, MB = Miami Beach, ST = Steph’s, SB = Struggle Bus, NURS = Nursery.

### Mortality

Coral mortality throughout the experiment was significantly influenced by site (Cox-Hazard, p<0.001) but not by genotype or the interaction of terms. In June, prior to the onset of bleaching, coral mortality was variable and significantly different by site (χ^2^ = 172.2, df = 7, p<0.01). Mortality ranged from 0% to 42% of outplanted colonies at Steph’s and Inshore, respectively. Notably, mortality in June was >30% at three sites (Inshore, Miami Beach, Struggle Bus) and <5% at the remaining 5 sites (Cooper’s, CVFD, Grounding, Jon’s, Steph’s), indicating that some sites are more challenging habitats post-transplantation than others. Genotype was not a significant driver of initial mortality (χ^2^ = 8.1, df = 7, p>0.05).

All mortality observed after the growth period was attributed to bleaching stress, though unobserved predation or disease could have contributed. At each timepoint, bleaching mortality was significantly influenced by site but not by genotype. In July, mortality was highly variable and significantly different by site (χ^2^ = 152.7, df = 7, p<0.01), ranging from 1% to 53% at CVFD and Inshore, respectively. In August, mortality was also site specific (χ^2^ = 128.1, df = 7, p<0.01), ranging from 8%-65% at CVFD and Struggle Bus, respectively. Recovery (% of surviving corals exhibiting normal coloration) was significantly impacted by site (χ^2^ = 82.7, df = 7, p<0.01) and genotype (χ^2^ = 41.4, df = 9, p<0.01), with nearly 80% of surviving corals coming from just three sites (Cooper’s Reef, Jon’s Reef, Miami Beach). In addition, there was a distinct bimodal pattern in recovery by genotype, with 6 genotypes at ~15% and 4 genotypes with ~1% survivorship. There was significantly higher recovery at the ‘cool’ sites compared to ‘hot’ sites (24% vs. 7.5%, t-test p = 0.0327). Neither pooled growth by site (r^2^ = 0.035, p = 0.656) nor pooled growth by genotype (r^2^ = 0.215, p = 0.176) predicted mortality. LE was not significantly different between corals that experienced subsequent morality and those that survived (Wilcoxon, p = 0.136).

## Discussion

*Acropora cervicornis* is an important reef-building coral in the Caribbean, forming habitat for many associated organisms [62] and structure needed for reef function [3, 63]. In recent years, it has become a focus for mitigation by active restoration to limit the extensive declines in this region [64]. The habitats and coral genotypes used here cover a broad environmental range of Florida reefs and expand on prior results showing differential growth among genotypes in a common garden [29, 30] and variable growth between sites [65]. We show *A*. *cervicornis* transplanted onto a variety of reefs exhibits variable growth and disturbance response dynamically dictated by multiple factors. Coral genotype was a significant factor in pooled growth, where environmental variability might be expected to overwhelm genotypic differences between individuals, meaning genotype is an important driver of colony success. Environment was also a significant determinant of growth, with reefs classified into three significant growth levels over a four-fold difference in average growth. In addition, extensive phenotypic plasticity in growth rate is evident for individuals in different environments, which may contribute to the apparent lack of local adaptation observed in this study.

The most influential driver of growth and survivorship in this experiment was the environment, though the impacts of specific metrics (temperature, carbonate chemistry, light, etc.) were difficult to resolve. Very shallow sites (~2m) had the lowest LE (CVFD and Steph’s), and sites with low light levels (regardless of depth) had significantly higher LE (Struggle Bus and Inshore reefs), suggesting that light at shallow sites may be so high that it is inhibitory [66]. Inshore and offshore reef sites in Florida show similar diurnal patterns in light, but significant cross-shelf and seasonal differences in carbonate chemistry and light [67, 68]. Given that six of the eight sites were ‘mid-channel’ (Table A in S1 File), it is not unexpected that there were no significant differences in Ω_arag_ between sites, as similar distance from shore suggests similar carbonate chemistry [68]. Depth is a primary driver of the diurnal variability in carbonate chemistry on reefs, with variance increasing with decreasing depth [69], yet variance in Ω_arag_ did not change with depth, and was actually lowest at one of the shallowest sites. More thorough sampling of carbonate chemistry and light (e.g., time-series), particularly the diurnal variability in Ω_arag_, is necessary to fully understand the role of these variables on growth, but were not resolvable here due to field constraints. The importance of genotype is further supported by the lack of significant relationship between LE and temperature, which is often the primary and most important environmental factor in coral growth [70]. Other abiotic variables not measured (e.g., nutrients, sedimentation) may also have influenced growth and survivorship.

Environmental characteristics were the primary driver of mortality throughout the growth period and had a strong influence on bleaching. The ‘challenging’ sites identified here (Struggle Bus and Inshore) and Miami Beach, a hardbottom habitat with high turbidity, showed high mortality during the first three months of this study, prior to bleaching. Contrary to previous work on the importance of high irradiance in bleaching response [71], patterns of bleaching between high and low light sites changed over time. Furthermore, no significant differences were found in bleaching prevalence between ‘hot’ and ‘cool’ sites. Importantly, certain sites where light regimes were significantly different but temperature was similar (e.g. Struggle Bus and Cooper’s), showed lower bleaching prevalence in the lower light environments, highlighting the importance of light as a factor in bleaching severity [71–73]. Overall, temperature differences between sites were most important during the recovery period, where a significantly higher proportion of corals transplanted to ‘cool’ reefs survived to the end of the experiment. Thus, small differences among sites influence survivorship and indicate the presence of potential habitat refugia, especially if novel combinations of coral genotypes are utilized. While these data are not representative of the response of all corals to all stressors, the occurrence of a natural disturbance provides strong context for understanding stress during bleaching. Survivorship during different types of disturbance, such as cold-water events [74] may show different patterns. The influence of the environment on *A*. *cervicornis* has important implications for future reefs in a changing climate. Varying conditions may lead to different population dynamics, particularly if changing population distribution involves range shifts that include marginal habitats [15, 75].

Coral genotype also significantly influenced growth rates, even when evaluated across a range of environments. Coral genotypes could be classified as fast or slow growing, but every individual varied >200% between the fastest and slowest growing locations, indicating broad acclimatization potential based on the host of conditions at a given site. Especially high growth differences between specific sites indicated that some individuals may be specialists that thrive only under certain conditions (e.g., cool temperatures, high turbidity, high light) and exhibit low relative growth rates elsewhere [51]. In contrast, the reaction norms of some genotypes showed a somewhat more consistent growth response, indicating they may play a more generalist role and have higher fitness over a broad range of environments [50]. For example, the Government Cut genotype, which had the highest average LE, can be considered a generalist, as it showed growth rates above the median at every site while maintaining one of the narrowest ranges in growth of any genotype. Conversely, Steph’s genotype represents a specialist, which is more dependent on environmental conditions and has higher growth variance; this genotype is the fastest growing coral at one site (CVFD) and the slowest growing coral at another (Jon’s). The development of these two strategies may be related to temporal environmental heterogeneity [76], where more variable environments produce generalist responses able to cope with temporal change. Generalist corals that are able to maintain growth at an even wider range of environments than those investigated here may be critical for long-term survival and recovery of diminished *A*. *cervicornis* populations, representing valuable additions to coral propagation and restoration programs in regions facing uncertain future conditions.

In addition to linear extension, genotypic differences in skeletal density and patterns of faster linear extension in corals with larger lipid content illustrate the various ways in which individual genotypes are unique, interacting with the environment. The tradeoff between high-density skeletons and fast growth found here is an important driver of intraspecific differentiation in fragmentation potential which may lead to distinct distributions on individual reefs, an example of phenotypic differentiation. Though no data was collected here, growth tradeoffs will influence long-term recovery trajectories, especially if reproductive capacity is compromised. Over the long-term, higher growth allows individuals to escape size specific mortality and will generally increase colony level fecundity due to size, but short-term sacrifices may also be important. Reproductive investment is among the first processes to be compromised during stress [77], so a better understanding of the balance between growth and reproductive output is critical for restoration. This tradeoff is particularly important if sexual reproduction can achieve a large recruitment event, jumpstarting species recovery. In this context, phenotypic plasticity provides practical information for understanding impacts of future global change on restoration[16, 78], illustrating the unique balance of factors dictating the response for reach genotype.

Genotype also influenced bleaching and mortality in this experiment, though more subtly than environmental conditions. Mortality was not different between genotypes until after the temperature maximum, but genotypic influence was apparent for the onset of bleaching prevalence in July. Bleaching patterns did not directly translate to mortality, likely because high temperatures overwhelmed genotypic differences and the ability of the holobiont to cope with the intense thermal challenge. Propagation in the common garden nursery can be expected to minimize (but not eliminate) maternal effects from the site of origin, so differential response to heat stress is attributed mostly to intrinsic differences in these corals and their symbionts, either through genetic predisposition, acclimatization after outplanting [50] or fine-scale differences in *Symbiodinium* communities and the microbiome [79]. Beyond maintenance in a single environment to minimize maternal effects, distinguishing between long-term acclimatization (i.e., to the original home site) and genetic differences is beyond the scope of this study. Though symbiont identity plays a critical role in growth and bleaching response [80, 81], all genotypes in this experiment contained only clade A upon outplanting. Any differential growth or bleaching response based on symbiont community must therefore be based on sub-cladal differences in thermotolerance [82, 83] or symbiont density [84].

Importantly, coral genotype was a determinant of survivorship after the bleaching event. In this case, temperature stress resulted in two groups of genotypes, one with approximately 15% survivorship and another with approximately 1% survivorship. The more robust individual genotypes with higher survivorship highlight the importance of GxE interactions and exemplify how habitat refugia may act synergistically with specific coral genotypes, increasing their ability to survive extreme disturbances. In this experiment, examples of GxE combinations that yielded high survivorship include Steph’s genotype transplanted at Cooper’s Reef (70% survivorship), Inshore genotype at Cooper’s (70%) and Inshore genotype at Jon's Reef (60%). Given that these sites experienced significant thermal stress (≥28 days over 31°C, maximum temperature >32°C), they represent disturbance resistant combinations. Multiple genotypes only survived at ‘foreign’ sites, illustrating how the environment can produce refugia for individual genotypes and the importance of spreading the risk of mortality of individual genets through restoration.

The integration of genotype and site affects can be seen in patterns of local adaptation, where foreign corals had higher pooled growth than native corals but no difference in survivorship, supporting the conclusion that individuals are not fine-tuned for specific habitats on the scale examined here. The lack of local adaptation observed here contrasts patterns observed in *Porites astreoides* [40]. Unlike *A*. *cervicornis*, *P*. *astreoides* is a brooding coral, with shorter time to competency, reduced dispersal, and vertical transmission of symbionts. These characteristics have the potential to reduce gene flow, promoting species-specific local adaptation patterns [40] and potentially driving interspecific differences in adaptive capacity. Local adaptation with respect to temperature has been demonstrated in Pacific species [21, 39], though environmental gradients in our experiment are likely more subtle, providing better context on the local scale. This result also conflicts with previous observations of genetic population structure over small spatial scales in *A*. *cervicornis*, which could be a sign of local adaptation on individual reefs, where genetic diversity is concentrated [49]. An experimental design which focuses on multiple genotypes per site may help resolve these differences, illustrating the functional impacts of local adaptation in this species.

Growth plasticity may act in an antagonistic manner to local adaptation, so observations of high plasticity may help explain the apparent lack of local adaptation. Another possible factor that would limit local adaptation is the mismatch between individual corals and their current environment, which could have changed and is no longer evolutionarily relevant. In addition, the analysis of local adaptation is limited by the random nature of genotype selection at the beginning of the experiment, where a single coral was selected from each reef. To address this, control and native genotypes (representing multiple colonies from a given reef) were pooled for analysis, expanding the genetic diversity of local types within each site, tempering this concern to some degree. In addition, the selection of large and healthy colonies for this experiment makes it more likely that we would find local adaptation if it existed, suggesting that our results are conservative. In addition, maintenance of corals in the common garden shared environment is designed to minimize the impacts of long-term acclimatization from the site of origin and isolate genetic affects[50]. Though this process is imperfect, it is the best available option for many marine organisms such as corals that are difficult to breed. It is also important to note that temporal affects may influence this result. Acclimatization to a new site may mean that relative growth rates change over time as individuals adjust to the environment, so it is possible that local adaptation would be evident over longer time scales. Seasonality should also be considered as different individuals may take advantage of changing conditions throughout the year and exhibit different growth patterns, which is especially important in the context of generalists and specialist[51].

One interesting trend documented is the low growth of nursery controls (i.e., control corals kept within the nursery site) and most wild controls, which were in the bottom 10% of growth rates at 85% of sites. Further, growth rates were lower for nursery controls of 80% of genotypes compared to average growth at all other sites. These trends suggest that changing environments during transplantation stimulates growth in fragmented corals, beyond the initial increase in growth rate or “pruning vigor” produced by fragmentation within a site [9]. This added growth after fragmentation and transplantation may be an adaptation to improve fragment cementation and survival in a new microhabitat, though these changes may come at the expense of skeletal density and involve other energetic tradeoffs.

Though the interaction term of the two-way ANOVA was not significant, data shows a dynamic relationship between specific combinations of site and genotype as would be expected during an interaction. High variability between sites and bleaching disturbance likely compromised the power to find a significant interaction if it exists, however this process is still potentially important. First, reciprocal differences in growth between sites (i.e. crossed reaction norms) for various genotypes, as shown extensively here, are evidence of GxE. Next, the absence of conserved reaction norms, which describe patterns of response across a range on conditions, provides evidence that site and genotype are flexibly interrelated, despite the non-significance of the interaction term. Lastly, ignoring interactive effects between these main factors masks the intrinsic flexibility of growth and survivorship patterns, providing a naïve explanation where site and genotype strictly dictate outcomes. The interaction between site and genotype highlight the subtle and complex interrelationship between factors that drives success and failure in this species.

A limitation of this study is the importance of *Symbiodinium* to the function of the holobiont, which recent evidence suggests is increasingly important. Analysis here shows that site, genotype and their interaction only explain 41% of variation in growth, so other factors including the microbiome likely play an important role. Acroporids are commonly associated with *Symbiodinium fitti* (type A3), but differentiation in function between strains of the same type [85, 86] can impact thermal tolerance and expression level, which are contingent on an interaction with the host [79]. We observe that the coral host significantly impacts growth rates and recovery after stress, however this affect is likely influenced by functional diversity of the sub-type symbiont strain, which we cannot resolve at the appropriate scale. By taking advantage of the ‘acroporid—*S*. *fitti* system’ [79], a better understanding of the impacts of these factors on growth and survivorship may be possible, but remains unresolved in this experiment. The microbiome may also play a functional role in growth and survivorship responses between genotypes shown here, but determining the relative contribution of these factors is difficult [83] and should be a focal point of future research.

Phenotypic plasticity suggests that *A*. *cervicornis* may be able to cope with changing conditions, up to a threshold, given the current levels of genetic diversity [87]. It is also possible that plasticity has helped maintain genetic diversity, which is still high in Florida [49, 88] during population declines on Caribbean reefs that might be expected to create a genetic bottleneck. Since different genotypes show unique reaction norms, emergent patterns of growth and survivorship may arise as new genotypes are matched to new environments, potentially allowing for human intervention to aid adaptation and acclimatization. The importance of cryptic genetic variation may become evident as these new combinations are discovered, pairing novel alleles and conditions leading to a range of phenotypes [35, 36]. Likewise, the evidence for some generalist genotypes implies that some individuals may be successful across a range of conditions. If the range in which generalists can grow into healthy colonies and the potential range of synergistic factors for specialists include future environmental regimes, phenotypic plasticity may spread the risk of mortality for certain genotypes across sites. By this mechanism, long-lived corals may serve as a bridge to sexual reproduction and subsequent adaptation, contributing to assisted gene flow through redistribution during restoration [89] which may help maintain diversity needed for reef survival under changing climate.

The apparent absence of local adaptation is also relevant for restoration. The fate of outplanted corals is determined by site and coral genotype, but low local adaptation indicates that individuals will not be systematically disadvantaged at new or foreign sites, at least over the spatial scales investigated here. Nursery repositories may therefore be able to supplement natural recruitment, since higher growth in foreign/immigrant corals counters some concern about particular traits that are uniquely suited to native environments. This barrier has been a concern for restoration [90], but should be tempered by our results. Maximizing genotypic (and thereby genetic) diversity should be a focal point of future outplanting efforts and will serve to produce novel GxE combinations and enhance reproductive success.

Overall, this experiment resolves a flexible system where coral genotype and environmental conditions contribute to the growth and survivorship response of a threatened species in a highly dynamic manner. Our data indicate that small environmental variation can drive ecologically important differences, especially in combination with specific coral genotypes, serving as refugia. Although population declines have been severe, phenotypic plasticity may help maintain population sizes under changing conditions, adding to the adaptive potential for change needed for long term sustainability of coral reefs.

## Supporting information

S1 File Supporting Figures and Tables. (PDF)

S2 File Supporting Results. (DOCX)

S3 File Data Deposit. (XLSX)
