# Supplementary material for: Genotype and local environment dynamically influence growth, disturbance response and survivorship in the threatened coral, Acropora cervicornis
Source: PLoS One. 2017 Mar 20;12(3):e0174000. doi: 10.1371/journal.pone.0174000 (PMC5358778; doi:10.1371/journal.pone.0174000)
Supplement: S1 File — (PDF) [file pone.0174000.s001.pdf]

Figure A- PAR values for four sites. Light readings represent hourly averages for four days at each site. Logistical constraints lead to sequential sampling, with Inshore and Coopers sampled immediately before CVFD and Struggle Bus.

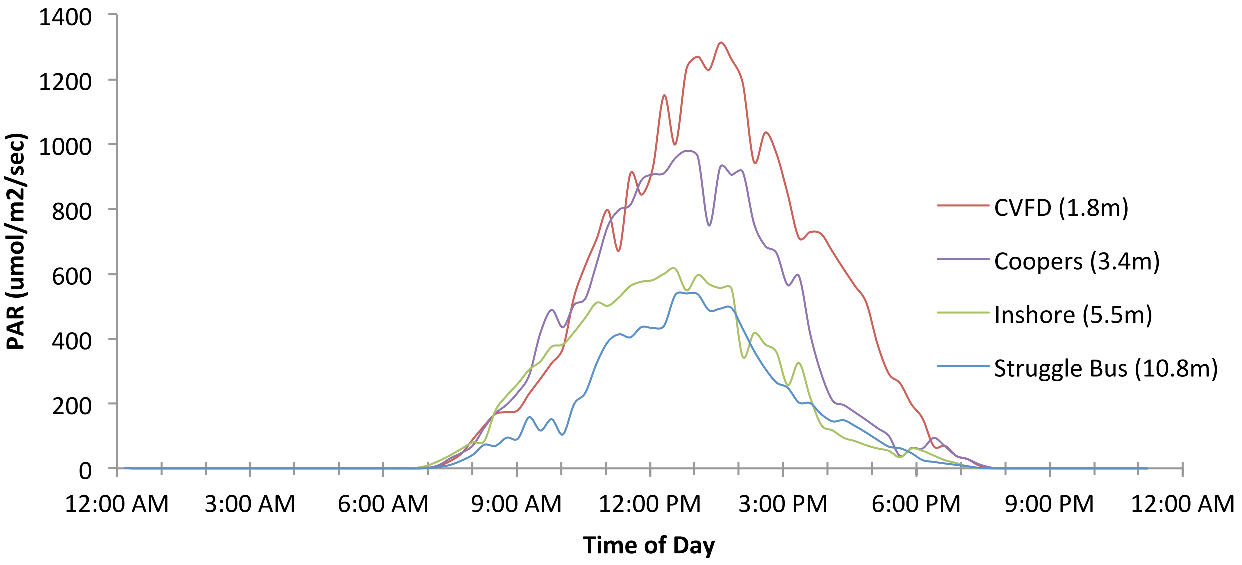

Figure B- Average temperature at all sites over time. Black line at 30C for reference. Bars represent sampling timepoints: installation, June, July, August, and December.

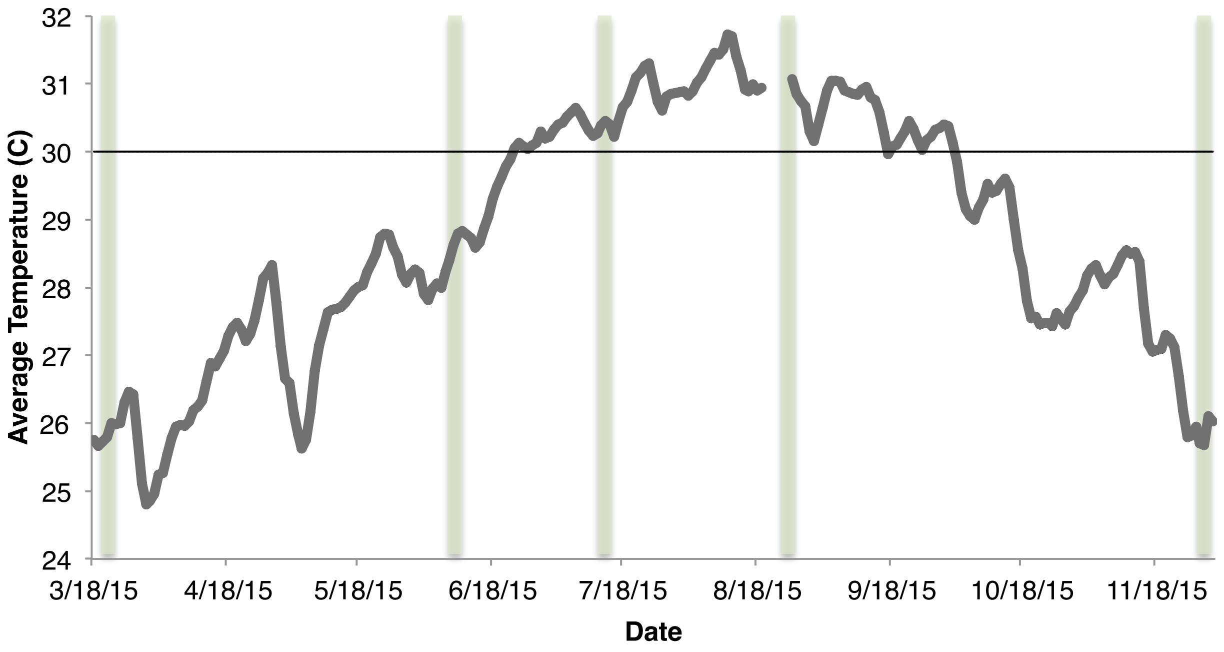

									Temperature								
									March - June Growth Period			July - August Bleaching Period			December Recovery Period		
**Site**	**Depth (m)**	**Lat**	**Long**	**Habitat**	**Inshore/Offshore**	**Collection/Outplant**	**Light**	**Average Ω**	**Average**	**Max**	**Days >31**	**Average**	**Max**	**Days >31**	**Average**	**Max**	**Days >31**
Cooper's Reef	3.4	25.50877	80.12053	High Relief Patch Reef	Midchannel	Both	High (600)	3.88	27.4	30.7	0	30.6	32.3	36	27.2	28.8	0
CVFD	1.8	25.32242	80.18228	Low Relief Patch Reef	Midchannel	Both	High (779)	3.85	27.7	31.9	4	31.0	33.4	50	-	-	-
Grounding	1.8	25.38876	80.16516	Consolidated Hardbottom	Midchannel	Both	High (nd)	4.10	28.5	31.4	5	30.9	32.8	43	-	-	-
Inshore	5.5	25.46478	80.15733	High Relief Patch Reef	Inshore	Both	Low (384)	3.93	27.9	31.8	7	31.0	32.8	42	-	-	-
Jon's Reef	3.7	25.56706	80.10806	Low Relief Patch Reef	Midchannel	Both	Low (nd)	3.88	27.4	30.7	0	30.6	32.3	28	27.4	28.8	0
Miami Beach	5.5	25.84830	80.10381	Consolidated Hardbottom	Midchannel	Both	Low (nd)	3.78	28.8	31.8	9	30.6	31.8	37	27.2	28.8	0
Steph's Reef	2.1	25.44133	80.13375	Low Relief Patch Reef	Midchannel	Both	High (nd)	3.95	27.3	31.9	2	31.1	33.4	70	27.4	29.9	0
Struggle Bus	10.7	25.33282	80.16505	Low Relief Patch Reef	Offshore	Both	Low (297)	3.82	27.4	30.6	0	30.7	31.8	42	27.5	28.7	0
Site 211	6.1	25.69232	80.09730	High Relief Patch Reef	Midchannel	Collection Only	-		-	-	-	-	-	-	-	-	-
Government Cut	7.6	25.75644	80.10006	High Relief Patch Reef	Midchannel	Collection Only	-		-	-	-	-	-	-	-	-	-
Bowl Nursery	5.8	25.48	80.11	Fixed-to-bottom Platforms	Offshore	NA	-		-	-	-	-	-	-	-	-	-

Table A– Descriptions of collection sites, outplant sites and the nursery. Physical data includes daytime light levels (PAR: µmol/m^2/sec), aragonite saturation state (Ω_{arag}), average and maximum temperature (°C) and days over 31°C during the growth, bleaching and recovery periods. 'nd' indicates no data.

Table B- Average growth (cm/day ±1S.E.) and Relative fitness of native and foreign corals at all sites. Growth rates (cm/day; see Fig. 3a) represent the difference between local (wild controls and collections originally from that specific site) and foreign (all other genotypes). Significance values are t-test between local and foreign corals. Relative fitness is calculated

Site	Local Growth (cm/day ±1S.E.)	Foreign Growth (cm/day ±1S.E.)	Relative Fitness (S)	p-value
Coopers	0.031 ± 0.005	0.030 ± 0.002	0.032	0.8525
CVFD	0.012 ± 0.002	0.021 ± 0.002	-0.444	0.0012*
Grounding	0.027 ± 0.003	0.030 ± 0.003	-0.132	0.8063
Inshore	0.017 ± 0.003	0.043 ± 0.004	-0.647	<0.0001*
Jons	0.019 ± 0.005	0.029 ± 0.002	-0.357	0.0757
Miami Beach	0.029 ± 0.008	0.025 ± 0.002	0.156	0.4321
Stephs	0.008 ± 0.001	0.011 ± 0.001	-0.353	0.0518
Struggle Bus	0.055 ± 0.010	0.041 ± 0.004	0.328	0.874

Table C- Mean (± 1 SD) carbonate chemistry values for outplanting sites. TA and DIC values are CRM corrected.

Site	n	**Salinity** (ppt)	**TA** (µmol kg^{-1})	**DIC** (µmol kg^{-1})	**pCO2** (µatm)	**Ωarag**
Cooper's Reef	4	34.03 ± 0.65	2373.3 ± 19.5	2034.1 ± 38.2	408.3 ± 36.8	3.89 ± 0.23
CVFD	5	34.20 ± 0.64	2364.8 ± 7.2	2027.4 ± 14.8	410.4 ± 24.3	3.85 ± 0.19
Grounding	4	34.40 ± 0.84	2357.6 ± 7.2	1998.1 ± 14.3	397.5 ± 45.1	4.10 ± 0.07
Inshore	4	34.07 ± 0.76	2368.8 ± 18.6	2024.6 ± 29.8	402.5 ± 40.9	3.93 ± 0.17
Jon's Reef	5	34.04 ± 0.86	2370.3 ± 21.8	2029.2 ± 51.7	408.8 ± 83.6	3.88 ± 0.33
Miami Beach	5	33.98 ± 1.14	2358.6 ± 18.6	2028.5 ± 26.9	418.5 ± 54.5	3.77 ± 0.23
Steph's Reef	5	34.28 ± 0.67	2359.2 ± 8.8	2012.8 ± 16.1	399.9 ± 20.2	3.95 ± 0.18
Struggle Bus	5	34.14 ± 0.68	2367.1 ± 7.4	2033.9 ± 13.7	425.6 ± 28.0	3.81 ± 0.15
