# Supplementary material for: Genotype and local environment dynamically influence growth, disturbance response and survivorship in the threatened coral, Acropora cervicornis
Source: PLoS One. 2017 Mar 20;12(3):e0174000. doi: 10.1371/journal.pone.0174000 (PMC5358778; doi:10.1371/journal.pone.0174000)
Supplement: S2 File — (DOCX) [file pone.0174000.s002.docx]

*Growth between Genotypes at Single Sites*

There were 2 significant genotype-growth levels identified at Jon’s Reef (ANOVA, F_(8,69)_ = 5.466, p<0.001) and 3 significant levels at Inshore (ANOVA, F_(10,42)_ = 6.453, p<0.001) (Table 2a). There were also significant differences between genotypes at CVFD (Kruskal-Wallis, H=32.023, p=0.001).

*Growth between Sites among Single Genotypes*

Based on Tukey’s HSD, there were 2 significant groupings of transplant reefs based on growth rates for the Struggle Bus genotype (ANOVA, F_(7,54)_ = 4.550, p=0.001) and 3 levels for the Coopers (ANOVA, F_(7,38)_ = 4.825, p=0.001), Government Cut (ANOVA, F_(6,53)_ = 4.859, p=0.001), Inshore (ANOVA, F_(7,49)_ = 4.309, p=0.001), and Steph’s (ANOVA, F_(7,43)_ = 4.884, p=0.001) genotypes (Table 2b). There were also significant differences in growth between transplant sites for the CVFD (Kruskal-Wallis, H=15.738, p=0.033), Site 211 (Kruskal-Wallis, H=21.863, p=0.003), and Jons Reef (Kruskal-Wallis, H=24.985, p=0.001).

*Local Adaptation/Selection coefficients*

By using growth as a fitness metric, selection coefficients were calculated to express relative fitness. Negative relative fitness at five sites ranged from -0.132 to -0.444; three sites showed positive fitness coefficients ranging from 0.032 to 0.328 (Table S1). Site CVFD showed the strongest negative relative fitness for native genotypes, partially driven by the growth of the lowest ranking genotype (Fig. 2b).

*Environmental Data*

There was no significant difference in aragonite saturation state (Ω) between sites for raw data (Kruskal-Wallis, H=6.54, p=0.477). Data was also adjusted to account for seasonal fluctuations (i.e. higher values in Spring) by taking the difference between individual saturation state values and the average within the timepoint to correct for seasonal fluctuations. These differences were averaged and showed no significance between sites (ANOVA, F_(7,11)_=0.2961, p=0.941). There was no significant relationship between growth and raw Ω (r^2^ = 0.026, p= 0.699) or seasonally corrected Ω (r^2^ = 0.039, p= 0.637). When skeletal density was used in combination with genotype specific growth rates to examine calcification, there were no significant differences between genotypes (ANOVA, F_(9,68)_=0.389, p=0.937)

CVFD (average daytime value over 4 days = 779 ±56 μmol/m^2^/sec; depth = 1.8m) and Coopers (600 ±49 μmol/m^2^/sec’ depth =3.4m) had significantly higher light than Inshore (384 ± 31 μmol/m^2^/sec; depth =5.5m) and Struggle Bus (297 ± 27 μmol/m^2^/sec; depth =10.7m) (Fig. S2). Maximum light levels ranged from 1313 μmol/m^2^/sec at CVFD to 539 μmol/m^2^/sec at Struggle Bus for any instantaneous reading.

Average, minimum, maximum, range, and variance of temperature were not significantly predictive of growth by site. Daily temperature range among all sites was typically around 1.5 °C, but mean temperatures were not significantly related to depth (r^2^=0.035; p=0.656).
